# LIFE ON THE STREETS IS HORRIBLE: OLDER RURAL-URBAN MIGRANTS COPE WITH HOMELESSNESS IN ETHIOPIA

**DOI:** 10.1093/geroni/igac059.2133

**Published:** 2022-12-20

**Authors:** Getachew Gebeyaw, Messay Kotecho, Margaret Adamek

**Affiliations:** University of Gondar, Gondar, Amhara, Ethiopia; Addis Ababa University, Addis Ababa, Adis Abeba, Ethiopia; Indiana University, Indianapolis, Indiana, United States

## Abstract

The prevalence of homelessness among older adults in Ethiopia is growing. While prior studies examined the push factors and challenges of homeless elders, little is known about how older people in Sub Saharan Africa cope with homelessness. This study explored the coping strategies of homeless older people in Kobo Town, Ethiopia. Purposive sampling was used to identify 10 homeless older people and four key informants. Study participants were homeless for a year or longer. Thematic analysis was used to analyze the data collected through in-depth interviews. To cope with the challenges faced on the street, homeless older people used various strategies including begging, holy water, drying leftover food, using river water for hygiene and sanitation, sleeping in church compounds, and creating their own social networks. Despite their efforts, the coping strategies used by elders were not sufficient. In the absence of family and government support, study participants relied heavily on begging to meet their survival needs. The findings call attention to the need for a national income support program and other supportive services for older adults. Homelessness is the product of a failing support system. Despite Ethiopia having a Plan of Action for Older Persons and Social Protection Policy for Vulnerable Groups, these policies have not been effectively implemented leaving older adults with no safety net. This study calls for the development of new policies to empower older people in Ethiopia and prevent them from turning to begging as their only recourse.

