# Correction to: LIFE ON THE STREETS IS HORRIBLE: OLDER RURAL-URBAN MIGRANTS COPE WITH HOMELESSNESS IN ETHIOPIA

**DOI:** 10.1093/geroni/igad026

**Published:** 2023-03-27

This is a correction to: Getachew Gebeyaw, Messay Kotecho, Margaret Adamek, LIFE ON THE STREETS IS HORRIBLE: OLDER RURAL-URBAN MIGRANTS COPE WITH HOMELESSNESS IN ETHIOPIA, Innovation in Aging, Volume 6, Issue Supplement_1, November 2022, Page 566, https://doi.org/10.1093/geroni/igac059.2133

In the originally published version of this abstract, affiliation 1 was erroneous and it has now been corrected to University of Gondar, Gondar, Amhara, Ethiopia.

